# The muscle – fat duel or why obese children are taller?

**DOI:** 10.1186/1471-2431-6-33

**Published:** 2006-12-13

**Authors:** Dina Ralt

**Affiliations:** 1 Izun & Tmura, Integrative Health Inst. 6 Nezach Israel st. Tel Aviv, 64352, Israel

## Abstract

**Background:**

Obesity the epidemic of our times appears to be a problem that is easy to resolve: just eat less and move more. However, this very common condition has turned out to be extremely troublesome, and in some cases even irreversible.

**Methods:**

The interplay between less muscle and more fat tissue is discussed from physiological perspectives with an emphasis on the early years of childhood.

**Results:**

It is suggested that the coordinated muscle-fat interactions lead to a fluctuating exchange economy rate. This bodily economic decision, slides between thrift (more fat) and prodigal (more muscle) strategies. The thrift strategy results not only in obesity and less physical activity but also in other maladies which the body is unable to manage.

What leads to obesity (less muscle, more fat) might be very difficult to reverse at adulthood, prevention at childhood is thus recommended.

**Conclusion:**

Early recognition of the ailment (low muscle mass) is crucial. Based on studies demonstrating a 'rivalry' between muscle build-up and height growth at childhood, it is postulated that among the both taller and more obese children the percentage of children with lower muscle mass will be higher.

A special, body/muscle-building gymnastics program for children is suggested as a potential early intervention to prevent the ill progress of obesity.

## Text

The Health Survey for England warns that by 2010, if nothing is done, 19% of boys and 22% of girls aged two to 15 will be obese [1]. Larger body size is not the main problem of obesity [2], the problems are the numerous accompanying maladies [3-5].

The spreading of the obesity epidemic, termed Globesity [6,7], is pointing to a very distressing situation – obesity not only seems irreversible but its incidence continues to rise. Though adenovirus showed association with obesity [8] more basic interactions point to a non-viral physiological complexity of gaining weight. The increased calorie intake and decreased physical activity, allied with obesity, appear to be the result of a bodily economic strategy rather than just the result of behavioral aberration [9].

What is the nature of this physiological strategy (i.e. exchange economy rates)? It has many attributes but this commentary will focus on the interplay between muscle and fat tissue. Interplay that leads the body to economic decisions fluctuating between thrift and prodigal strategies. Consider for example, thermogenesis [10] or Non Exercise Activity Thermogenesis [11] as indirect parameters for such an exchange economy rate.

Muscles are amongst the costliest tissues of the body to maintain [12-14], therefore under certain conditions muscle 'deficiency' could be beneficial (conserving muscle energy may be advantageous, as the saved energy can actuate other functions). It is thus no wonder that built-up muscles rapidly revert to their original size unless trained or used constantly [15-17]. Muscle activity induces prodigal strategy which enables not only its working but also allows other bodily functions to operate, resulting in good health and positive mood. On the other hand fat accumulation occurs when food shortage is being expected or when excess food makes physical activity unnecessary. Fat accumulation needs extra available energy thus arresting energy expenditure is always associated with the weight increase. The more fat tissue available, the greater is its power to induce thrift strategy resulting not only in obesity and in decline of physical activity but also in other untreated health problems.

The aim of this commentary is to exhibit the contradictory physiological effects of the muscle and the fat tissue and to show how their integrated capacities determine our wellness.

Please note that fat accumulation will naturally overcome muscle buildup as without additional effort, access fat but not access muscle will be maintained. Overabundance of food was (until recently) rare and thus it is no wonder that no evolutionary tactics were developed to handle health mischiefs of excess food or lack of physical activity. Our natural tendency (based on long history of food shortage) is to save energy whenever possible, for example in walking we naturally choose the pace that minimizes energy cost per distance, even when this strategy requires a greater relative aerobic effort [18] (see also next paragraph).

Experiments with animals have shown that even the expression of a genetic predisposition to high physical performance strongly depends upon the environment experienced early in life. Fully fed lizards experienced a marked reversal of performance within only one month after birth [19]. The lizards take advantage of the excess food and save muscles activity. On the other hand, mice pups whose mothers had received 30% less energy during pregnancy were born underweight. These pups experience the increase in blood leptin a week earlier than usual, and when they receive a high-fat diet they gain weight faster than their counterparts born to normally-fed mothers [20]. These results indicate the ease with which fat is favored over muscle in the animal kingdom. The same trends of energy savings (less muscle, more fat) under deprivation were also observed in humans. Babies who were conceived during the Dutch famine of 1944–1945 showed higher rates of obesity at age 19 and age 50 than the rates of those conceived before or after that challenging period [21]. This suggests that babies who receive poor nutrition in the womb, 'expect' to face food shortages after birth as well, and their metabolism will be regulated to be especially thrifty with the calories they receive. When such individuals eat the rich diets typical of today's developed countries, they quickly become overweight. This obesity, which is part of the natural developmental plasticity [20], becomes inevitable and accentuated with lower muscle mass. Lower muscle mass can be the result of lack of physical activity, genetics and/or overeating and is observed in the more rapid growth of nowadays children. Indeed averaged body mass index is higher in tall than in short children (7, 23, 24). This is the opposite of a higher muscle mass condition, which results in slower growth rate. Gymnasts for example, experience growth spurts in height that occur approximately 1–2 years later than non-athletic adolescents [25-27]. The correlation between obesity and height has been established in many countries. A French study showed that the increase in the prevalence of obesity is indeed accompanied by a global trend of accelerated growth [28] and a study in Chile has shown that obese preschool children were four centimeters taller than the normal weight children [29]. The interplay between growth and obesity represents a physiological adaptive trait. This trait enables the body to choose between investing energy in growth or saving energy for storage.

Obesity hormones indeed do affect growth. It has been shown that tall and obese children exhibit variation in the ghrelin gene [30], and leptin was shown to stimulate growth even in the presence of caloric restriction [31].

## What can be done?

The ease with which many individuals gain weight suggests that the energy homeostasis system in the body is inherently biased toward weight gain [32] and thrift strategy. Moreover, in many mammals, energy stored in adipose tissue is held relatively constant [33]. These and other findings, like the many-fold regulatory materials [34,35] excreted by fat cells, suggest that fat cells are not inert fat storage depots but rather active, manipulative cells in 'dialogue' with muscles and other tissues [36,37]. These fat tissue tactics may be a burden to any obesity solution, and are especially severe for those with low muscle mass and hence less 'muscle-negotiation' capability. Avoiding fat storage as early as possible, before it starts to induce further fat accumulation is mandated [38]. The difficulty in losing weight was stated by the U.S. Preventive Services Task Force, which found insufficient evidence for the effectiveness of behavioral counseling or other preventive interventions with overweight children [39].

A special effort should be directed toward defining the children with initial lower muscle mass as their obesity might be very difficult to combat later. Besides tallness and obesity it may be interesting to look for additional parameters such as ghrelin variations [30], bone density [40] or a measure of diet-induced thermogenesis [41] that might discern the lower muscle mass group within the general obesity group.

As suggested, it is the muscle activity that can avoid a very difficult to reverse obesity and ill health, indeed the underappreciated role of muscles in health and disease has already been observed [42]. Not very many studies have directly tested the great benefits of exercise on children [43]. However, studies concerning strength training are starting to accumulate [44-47]. Such a recent study showed that obese children who participated in a weight-training group, experienced a significant increase in muscle strength, while the control group had no such increase. In the same study, the fat mass of the children in the weight-training group did not change during the ten-week course of the study, while the children in the control group gained an average of more than 2.5 pounds of fat during the same period [48].

If muscles are the main health negotiators with the fat tissue, a body/muscle-building gymnastics program for children, is required to help prevent the ill progress of obesity. Investigations to determine the influence of resistance exercise on child obesity are thus needed. As stated, such studies are indeed starting to accumulate and some, more intricate, reveal interesting results. In young women for example, it was shown that resistance exercise increased a spectrum of growth hormone molecules [49].

The resistance exercise approach can prevent or decrease weight gain early, helping all obese children and especially those with low muscle mass.

How about starting to think on muscles [50]?

## Pre-publication history

The pre-publication history for this paper can be accessed here:


